# A Comparative Study on Johnson Cook, Modified Zerilli–Armstrong, and Arrhenius-Type Constitutive Models to Predict Compression Flow Behavior of SnSbCu Alloy

**DOI:** 10.3390/ma12101726

**Published:** 2019-05-27

**Authors:** Tongyang Li, Bin Zhao, Xiqun Lu, Hanzhang Xu, Dequan Zou

**Affiliations:** 1 College of Power and Energy Engineering, Harbin Engineering University, Harbin 150001, China; lty123sy@163.com (T.L.); luxiqun@hrbeu.edu.cn (X.L.); xuhanzhang@hrbeu.edu.cn (H.X.); zoud@wusm.wustl.edu (D.Z.); 2 School of Medicine, Washington University in St. Louis, St. Louis, MO 63108, USA

**Keywords:** SnSbCu alloy, flow behavior, Johnson–Cook model, modified Zerilli–Armstrong model, Arrhenius-type model

## Abstract

The flow behavior of the SnSbCu alloy is studied experimentally by the compression tests in the range of the strain rates from 0.0001 to 0.1 s^−1^ and temperature from 293 to 413 K. Based on the experimental data, three constitutive models including the Johnson–Cook (J–C), modified Zerilli–Armstrong (Z–A), and Arrhenius-type (A-type) models are compared to find out an optimum model to describe the flow behavior of the SnSbCu alloy. The results show that the J–C model could predict the flow behavior of the SnSbCu alloy accurately only at some specific strain rates and temperature near the reference values. The modified Z–A and A-type constitutive models can give better fitting results than the J–C model. While, at high strains, the predictive values of the modified Z–A model have larger errors than those at low strains, which means this model has limitations at high strains. By comparison, the A-type model could predict the experimental results accurately at the whole strain range, which indicates that it is a more suitable choice to describe the flow behavior of the SnSbCu alloy in the focused range of strain rates and temperatures. The work is beneficial to solve the tribological problem of the bearing of the marine engine by integrating the accurate constitutive model into the corresponding numerical model.

## 1. Introduction

The SnSbCu alloy are preferred as the surface material in engine bearings for its good compliance, embeddedness, and anti-seizure property [1,2,3]. In actual working condition, the surface of bearing bears high temperature and pressure, especially in the marine engine main bearing, which the authors focus on. According to the research [4,5,6,7], it can be known that the specific pressure of the main bearing caused by the oil pressure and asperity contact ranges from tens of MPa to several hundred MPa (usually less than 300 MPa), and the temperature of the bearing shell is maintained between 323 K and 423 K. For most existing research studies about the bearing tribological performance, the material properties are usually considered as the constants under the operating condition [8,9,10]. However, as some research studies indicated in another field [11,12,13], the material constants would change since the flow behavior is influenced by three factors: strain, strain rate, and temperature. Metasch et al. [14] used the experiment to determine the SnSbCu properties for the Chaboche model. To describe the tribological properties of bearings more accurately, a more precise constitutive model should be obtained by first considering the effect of these three factors on the flow behavior of the SnSbCu alloy. However, it could be known from existing references that the related study on the SnSbCu alloy seems to be very limited, and more efforts are needed.

Several constitutive models have been proposed previously to describe the flow behavior for different materials, which could be divided into three categories: the empirical [15,16,17,18], phenomenological [19,20,21,22], and physical [23,24] models. The physical models like the Mechanical Threshold Stress (MTS) model and the Bammann–Chiesa–Johnson model were used for describing the flow behavior in many materials due to their high precision [25,26]. However, the physical model requires more data from the high-quality equipment to calculate many material coefficients. More often, the models with an appropriate number of material constants from limited experimental results (e.g., the empirical models) are preferred to describe the flow behavior, which could be more easily integrated into the further analysis numerical model. Regarding the empirical model like the J–C [27] and Z–A model, these models have been successfully used to predict the flow behavior of different materials and applied to the finite element (FE) model due to their high accuracy and simple forms. Duan et al. [28] established an FE model about the recrystallization in a white layer to simulate the day hard cutting process based on the J–C model. Laakso and Niemi [29] evaluated the performance of AISI 1045 for different J–C constants from the method combined with inverse analysis with an FE model and cutting experiments. Gurusamy and Rao [30] implemented a constitutive relationship established by modifying the Z–A model into an FE model of orthogonal machining process. Paturi et al. [31] used FE simulations to validate the modified J–C and modified Z–A model during orthogonal cutting processes. Khan and Liu [32] incorporated an A-type rate equation into a 3D crystal plasticity formulation implemented into the FE program. Anoop et al. [33] presented a microstructure-sensitive FE simulation approach, which considered the retained austenite using a modified A-type equation. To adapt to different materials, there have been many modified forms for the J–C and Z–A model in research studies [16,34,35,36,37]. Among the phenomenological models, they have been applied to represent the complex flow behavior in many materials because these models also have high precision with a limited number of material constants. However, phenomenological models do not involve micro-mechanism of material deformation, but only consider the effect of macro-deformation parameters (deformation temperature, strain rate, and strain) on flow stress. Therefore, different types of models have their own advantages and are suitable for different materials. When determining which kind of constitutive model is suitable for one specific material, a comparative study should be taken to contrast the accuracy and adaptability [38,39,40].

The J–C model is one of the most classical constitutive models and only has five material constants. Therefore, the material constant could be obtained by only a few experimental data points. Due to its high precision, simple form, and wide applicability, this model is still widely applied nowadays, especially in the finite element analysis. Shrot et al. [41] presented a method to determine constants of the J–C model from machining simulations to describe the material behavior during the high speed cutting process. Deng et al. [42] derived the J–C model of commercial pure (CP) titanium Gr2 at a high strain rate by quasi-static and dynamic compression tests. The J–C model has been proven to apply for a variety of materials under the wide range of temperature and strain rate.

The modified Z–A model, which is based on the Z–A model, has the complex relation among the influence factors. It is suitable to predict the flow behavior of a large amount of materials at different strain rates and temperatures, which range from room temperature to 0.6*T*_m_ (*T*_m_ is the melting temperature). Baghani et al. [43] considered the temperature-dependent behavior and used a modified Z–A model to describe the plastic hardening characteristics over a wide range of temperature. Mirzaie et al. [44] modified the Z–A model by taking into account both a hardening and a softening effect to predict the hot flow stress of materials. Lee et al. [45] chose the Z–A constitutive model to represent the plastic behaviors of the different carbon steel materials at a high strain rate to research the effect of carbon content on mechanical responses.

As a phenomenological model, the A-type constitutive model has been widely used in predicting material flow behavior, especially at high temperature. Lin et al. [46] and Mandal et al. [47] improved the strain-dependent hyperbolic sinusoidal constitutive model by compensating the strain rate in the Zener–Hollomon parameter to predict the deformation behavior at a high temperature for the 42CrMo and alloy D9 separately. Niu et al. [48] used the A-type constitutive model to take the incorporated influence of the strain rate and temperature into account by the experimental data to describe the compression deformation behavior of lead-free solders. Wang et al. [49] researched the deformation behavior of 20Cr2Ni4A, which is a high-strength alloy, at different temperatures and strain rates by the A-type model. Wang et al. [50] studied the hot deformation behavior of the Nickel-based corrosion-resistant alloy by establishing its the A-type model over the strain rate range of 0.001–1 s^−1^ at a high temperature.

From the mentioned studies, it could be seen that different constitutive models are suitable for different materials at the different range of temperatures and strain rates to describe the flow behaviors. In this study, the appropriate constitutive model of the SnSbCu alloy material is obtained. The stress-strain curves of the SnSbCu alloy are drawn experimentally by the electronic universal material testing machine under the isothermal temperature condition within a specific range of the strain rate. Three models, the J–C, modified Z–A, and A-type models, are used to predict the flow behavior. By comparison with the experimental results, the suitability of the three models is evaluated by calculating the correlation coefficient and average absolute relative error.

## 2. Experiment

**Figure 1:**
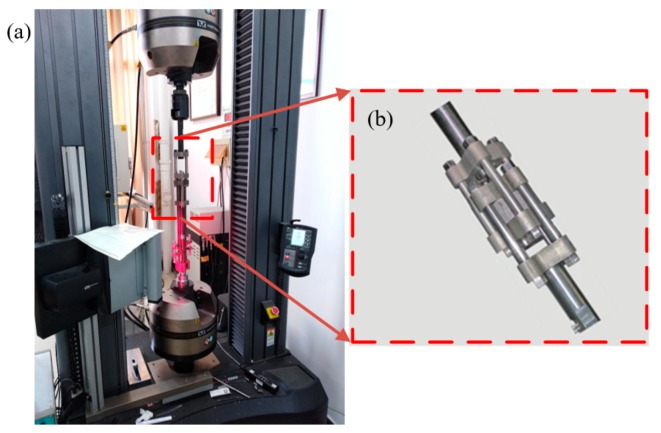
Experimental equipment for the quasi-static tests (**a**) Instron 5985 material universal testing machine and (**b**) fixture device.

The Instron 5985 material universal testing machine (Instron Worldwide Headquarters, Norwood, MA, USA, as shown in Figure 1a) was used to measure the quasi-static stress-strain curve of the alloy at the strain rate of 0.0001 s^−1^, 0.001 s^−1^, 0.01 s^−1^, and 0.1 s^−1^ under different temperatures (293 K, 323 K, 353 K, 383 K, and 413 K). Cylindrical specimens with 9 mm in height and 6 mm in diameter were prepared for the quasi-static tests. The experiment was carried out in an environmental chamber to ensure that the actual experimental temperature was the same as the preset temperature. The experimental temperature was raised to the specified value and held for 15 min to assure that the sample reached the preset temperature and was compressed at a certain strain rate.

In this experiment, it is worth noting that the fixture (as shown in Figure 1b) is used to replace the compression with the tension. The fixture consists two symmetrical and non-interfering platforms. The sample is placed between two platforms. When the test machine performs the pulling force on the fixture, the pulling force is transmitted to pressure between the platform and sample. To reduce deviations from the material universal testing machine, the video extensometer and infrared thermometer were applied to correct the deviation of the strain and temperature, respectively, during the experiment. Three parallel experiments at each temperature and strain rate were performed to guarantee the accuracy of the experimental data.

## 3. Results and Discussions

### 3.1. Experimental Results

**Figure 2:**
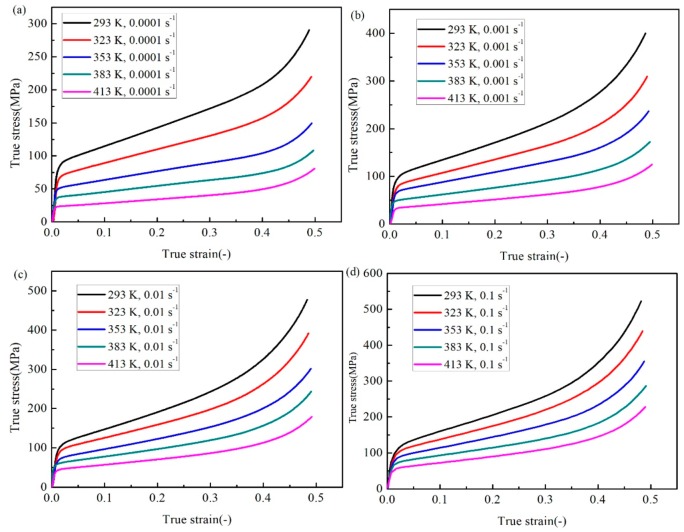
The true strain-stress experimental results for different temperatures and the strain rate of (**a**) 0.0001 s^−1^, (**b**) 0.001 s^−1^, (**c**) 0.01 s^−1^, and (**d**) 0.1 s^−1^.

All the experimental results have been shown in Figure 2. It shows that the true stress increases with the rise of the true strain at a specific temperature and strain rate as expected, while it decreases significantly at the same strain as the temperature changes in a specific strain rate case, which means the temperature has a strong effect on the flow behavior of the SnSbCu alloy. The effect of the strain rate on true stress is also considered and it indicates that, as the strain rate grows, the true flow stress goes up, which shows a notable strain rate hardening effect. In addition, the stress gap between two strain rates gets larger as the strain rate becomes smaller, which demonstrates that the hardening effect enhances at smaller strain rates. From Figure 2, it could be clearly found that the flow behavior cannot be simply considered since the linear superposition of the strain rate hardening effect and temperature softening effect. Thus, a constitutive model, which could consider the effects at the same time should be selected to describe and predict the flow behavior of the SnSbCu alloy precisely under the focused ranges of strain rates and temperatures. In these experiments, the material would be the densification stage when the strain is bigger than 0.4. At this stage, the flow stress of the material increases rapidly with raising the strain. The plastic deformation stage of the material is the authors’ concern, so the data would not be considered when the strain is bigger than 0.4.

### 3.2. Johnson–Cook Model

**Figure 3:**
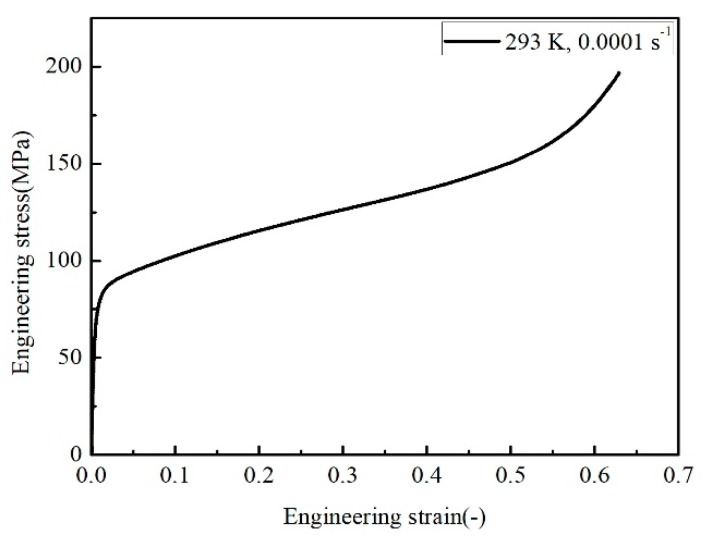
Engineering stress-strain curve at the reference condition.

The J–C model is used frequently to describe the relation between the stress and the strain of materials under a wide range of strain rate and temperature due to the simple form. The equation of the model [27] is expressed as follows.

(1) $$\sigma =\left(A+B\varepsilon^{n}\right)\left(1+C_{1}\ln{\dot{\varepsilon }}^{*}\right)\left(1-T^{*}{}^{m}\right)$$

where *σ* is the von Mises equivalent flow stress and *ε* is the equivalent plastic strain. ${\dot{\varepsilon }}^{*}$ is the dimensionless strain rate, getting from ${\dot{\varepsilon }}^{*}=\dot{\varepsilon }/\;{\dot{\varepsilon }}_{ref}$ where $\dot{\varepsilon }$ is strain rate and ${\dot{\varepsilon }}_{ref}^{*}$ is the reference strain rate. T* = (*T − T_ref_*)/(*T_m_ − T_ref_*) is the relative temperature. Where *T* is the experimental temperature, *T_ref_* is the reference temperature, and *T_m_* is the melting temperature. The melting point of the SnSbCu alloy focused in this work is 573 K, and the values of *T_ref_* and ${\dot{\varepsilon }}_{ref}$ are 293 K and 0.0001 s^−1^ separately. The meaning of the constants are as follows. *A* is the yield stress at a reference strain rate and reference temperature, *B* is the material hardening coefficient, *n* is the material strain hardening index, *C*_1_ is the strain rate sensitivity coefficient, and *m* is the temperature softening index. The yield stress could be calculated from the engineering curve of stress and strain under the reference strain rate and temperature directly. The engineering stress-strain curve is shown in Figure 3 at a reference strain rate and temperature, so *A* is equal to 89.96 MPa for the kind of alloy material and the other constants could be obtained from experimental data under some conditions, which is shown as follows.

When *T* = *T_ref_* and $\dot{\varepsilon }={\dot{\varepsilon }}_{ref}$ the terms of $\left(1+C_{1}\ln{\dot{\varepsilon }}^{*}\right)$ and (1 − T*^m^) are eliminated. Equation (1) could be reduced to the formula below.

(2) $$\sigma =\left(A+B\varepsilon^{n}\right)$$

Taking the natural logarithm, the equation is rewritten as follows.

(3) ln(σ−A)=nlnε+lnB

The values of *n* and *B* could be obtained from the relation between ln(*σ*−*A*) and ln*ε*. The slope (*n*) and the intercept (ln*B*) equal 1.107 and 312.4 MPa, respectively. When the deformation temperature is the reference temperature, Equation (1) could be simplified into the equation below.

(4) $$\frac{\sigma }{A+B\varepsilon^{n}}=1+C_{1}\ln{\dot{\varepsilon }}^{*}$$

**Figure 4:**
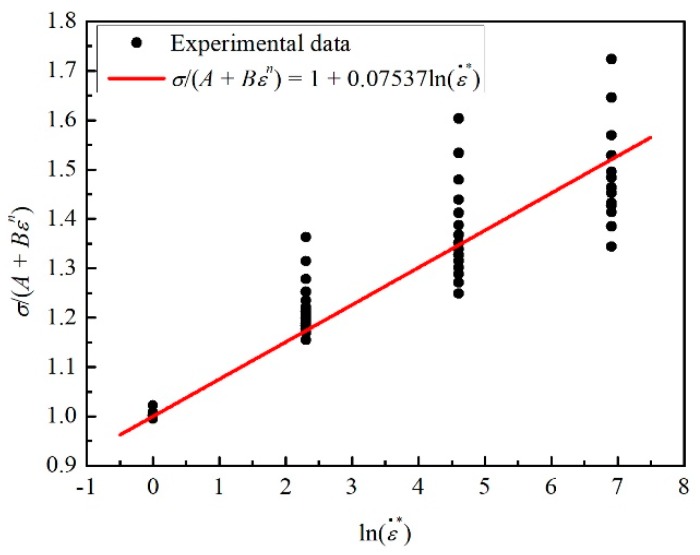
The relation between *σ*/(*A + Bε^n^)* and $\ln{\dot{\varepsilon }}^{*}$.

Sixty values of stress related to 15 strain values (0.05, 0.075, 0.1, 0.125, 0.15, 0.175, 0.2, 0.225, 0.25, 0.275, 0.3, 0.325, 0.35, 0.375, and 0.4) at four different strain rates of 0.0001 s^−1^, 0.001 s^−1^,0.01 s^−1^, and 0.1 s^−1^ were selected to draw the curve of *σ*/(*A + Bε^n^)* vs. $\ln{\dot{\varepsilon }}^{*}$. In addition, the value of *C*_1_ = 0.07537 could be acquired from the fitting curve, as shown in Figure 4.

When $\dot{\varepsilon }=\;{\dot{\varepsilon }}_{ref}$, which means ${\dot{\varepsilon }}^{*}$ = 1, the equation is the following.

(5) $$\ln\left(1-\frac{\sigma }{A+B\varepsilon^{n}}\right)=m\ln T^{*}$$

**Figure 5:**
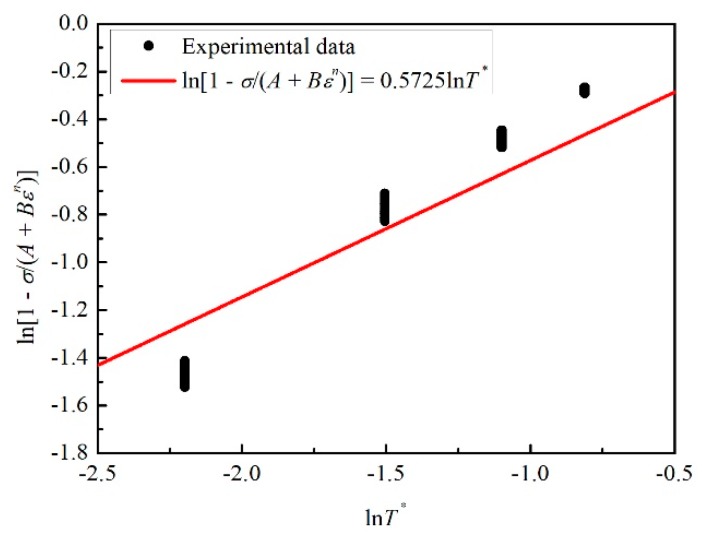
The relation between ln(1 − *σ*/(*A* + *Bε^n^*)) and lnT*.

Similarly, the cases of 15 identical strains at four different temperatures of 323 K, 353 K, 383 K, and 413 K were chosen to draw the relation between ln(1 − *σ*/(*A* + *Bε^n^*)) and lnT*. Additionally, the value of *m* could be obtained as 0.5725 by fitting the corresponding black points as shown in Figure 5.

The complete formula of the J–C model could be obtained as follows.

(6) $$\sigma =\left(89.96+312.4\varepsilon^{1.107}\right)\left(1+0.07537\ln{\dot{\varepsilon }}^{*}\right)\left(1-T^{*}{}^{0.5725}\right)$$

**Figure 6:**
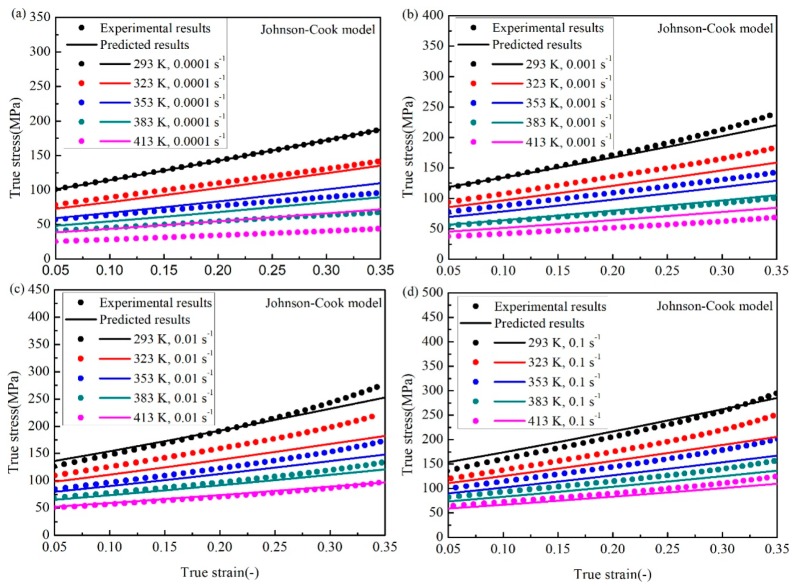
Comparison between the experimental data and the predicted values by the J–C model at the strain rate of (**a**) 0.0001 s^−1^, (**b**) 0.001 s^−1^, (**c**) 0.01 s^−1^, and (**d**) 0.1 s^−1^.

Using the constitutive model above, the flow stress of the SnSbCu alloy could be predicted at different temperatures and strain rates. The comparison between experimental results and the predicted results are shown in Figure 6. It could be observed that the predicted results from the J–C model could only match well with the experimental data points at the reference temperature and the strain rate. The main reason is that the strain rate hardening effect and the temperature softening effect are considered as two independent factors for the J–C model. However, as many researchers [11,12,13] indicated, the interaction between these two effects exists for some metallic materials. The interaction might also exist for the SnSbCu alloy.

### 3.3. Modified Zerilli–Armstrong Model

As mentioned before, to consider the interaction between the effect of the strain rate and temperature, a modified Z–A model is selected, which is expressed below.

(7) $$\sigma =\left(C_{1}+C_{2}\varepsilon^{n}\right)\exp\left[-\left(C_{3}+C_{4}\varepsilon \right)T^{*}+\left(C_{5}+C_{6}T^{*}\right)\ln{\dot{\varepsilon }}^{*}\right]$$

The meanings of σ, ε, and ${\dot{\varepsilon }}^{*}$ are the same as those in the J–C model. The difference is that the expression of T* is T* = *T* − *T_ref_* in this model. In this case, *T_ref_* = 293 K and ${\dot{\varepsilon }}_{ref}$ = 0.0001 s^−1^. Meanwhile, *C*_1_, *C*_2_, *C*_3_, *C*_4_, *C*_5_, *C*_6_, and *n* are material constants. *C*_1_ means the yield stress at the reference strain rate and reference temperature, which equals to 89.96 MPa, as obtained in the J–C model. Like the J–C model, the data under specific conditions was chosen to eliminate terms in the model and the constants could be obtained from the fitting curve, respectively.

First, taking the natural logarithm on both sides of Equation (7) into account, the equation could be represented below.

(8) $$\ln\sigma =\ln\left(C_{1}+C_{2}\varepsilon^{n}\right)-\left(C_{3}+C_{4}\varepsilon \right)T^{*}+\left(C_{5}+C_{6}T^{*}\right)\ln{\dot{\varepsilon }}^{*}$$

When the strain rate equals the reference strain rate, Equation (8) is simplified below.

(9) $$\ln\sigma =\ln\left(C_{1}+C_{2}\varepsilon^{n}\right)-\left(C_{3}+C_{4}\varepsilon \right)T^{*}$$

**Figure 7:**
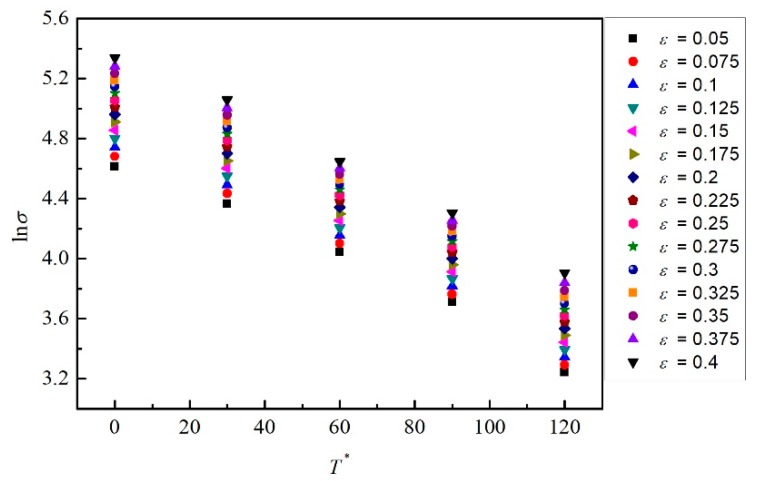
The relations between ln*σ* and *T* * for 15 different strains.

Under different temperatures, 15 strains (0.05, 0.075, 0.1, 0.125, 0.15, 0.175, 0.2, 0.225, 0.25, 0.275, 0.3, 0.325, 0.35, 0.375, and 0.4) and the corresponding stress values were chosen to describe the relation between ln*σ* and T^*^. As shown in Figure 7, the slope *S*_1_ and intercept *I*_1_ at different strains could be obtained, which are −(*C*_3_ + *C*_4_*ε*) and ln(*C*_1_ + *C*_2_*ε^n^*), respectively. Equations (10) and Equation (11) are shown below.

(10) $$I_{1}=\ln\left(C_{1}+C_{2}\varepsilon^{n}\right)$$

(11) $$S_{1}=-(C_{3}+C_{4}\varepsilon )$$

According to the two equations above, 15 couples of *S*_1_ and *I*_1_ are calculated from Figure 7, as shown in Table 1.

**Figure 8:**
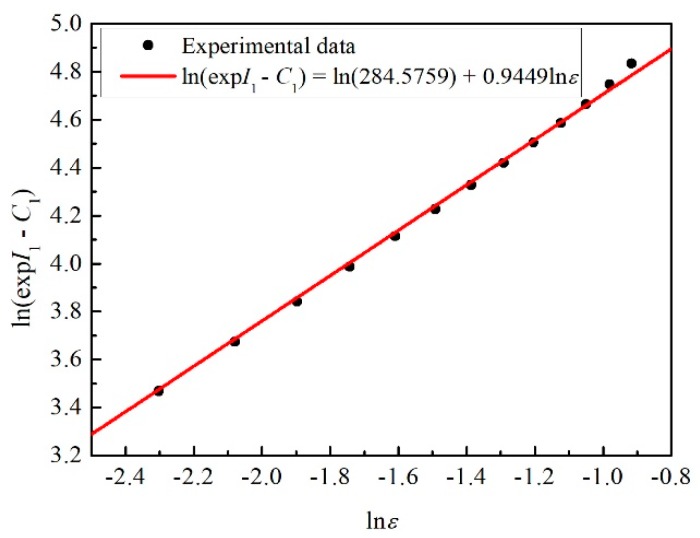
The relation between ln(exp*I*_1_ − *C*_1_) and ln*ε*.

By rearranging Equation (10), Equation (12) can be obtained. Afterward, the relation between ln(exp*I*_1_ − *C*_1_) and ln*ε* could be established, according to the values of *I*_1_ under different strains. As shown in Figure 8, ln*C*_2_ is the slope of the curve and *n* is the intercept. By calculation, the values of *C*_2_ and *n* are 284.5759 MPa and 0.9449, respectively.

(12) $$\ln\left(\exp I_{1}-C_{1}\right)=\ln C_{2}+n\ln\varepsilon$$

**Figure 9:**
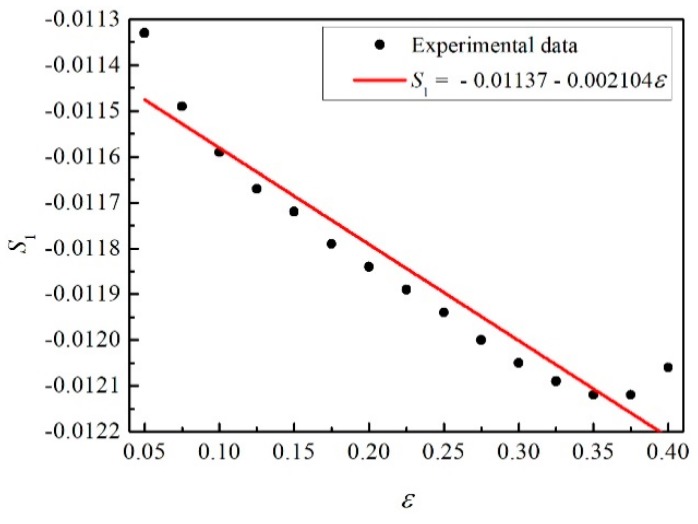
The relation between *S*_1_ and *ε.*

Moreover, the curve of *S*_1_ vs. *ε* is also plotted (shown in Figure 9). −*C*_3_ and −*C*_4_ are the values of intercept and slope in the fitting line separately, whose values are 0.01137 and 0.002104, respectively.

From Equation (9), the curves of ln*σ* vs. $\ln{\dot{\varepsilon }}^{*}$ can be plotted by the selected data under five temperatures (293 K, 323 K, 353 K, 383 K, and 413 K) and 15 strains, which are the same as those in Figure 7. Similarly, the value of (*C*_5_ + *C*_6_*T* ^*^) under different strains could be calculated as the slope *S*_2_. *S*_2_ can be expressed as the following.

(13) $$S_{2}=C_{5}+C_{6}T^{*}$$

**Figure 10:**
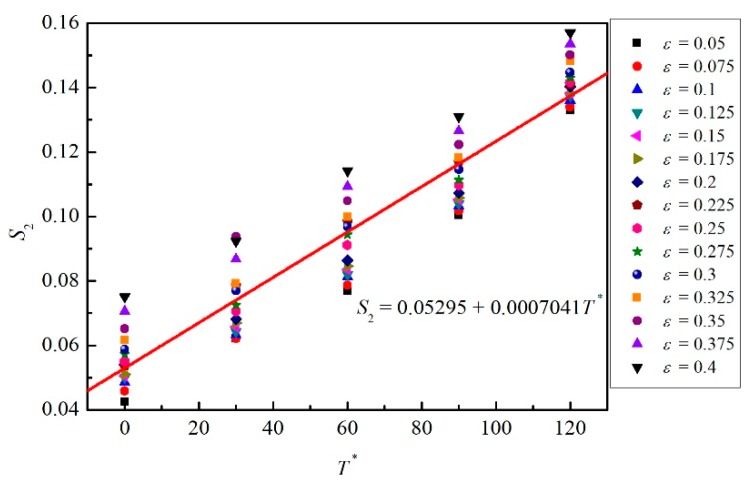
Scatter plot of *S*_2_ vs. T* for different strains and the most suitable fitting line.

Therefore, the values of *C*_5_ and *C*_6_ can be obtained by the relation between *S*_2_ and T*. As shown in Figure 10, 75 data points of *S*_2_ are used to fit the line, according to the minimum related error. Therefore, the value of *C*_5_ is 0.05295 and the value of *C*_6_ is 0.0007041.

Lastly, substituting the determined constants into Equation (7), the modified Z–A constitutive model of the SnSbCu alloy can be written using the equation below.

(14) $$\begin{array}{ll} \sigma =\left(89.96+284.5759\varepsilon^{0.9449}\right) & \exp[-\left(0.01137+0.002104\varepsilon \right)T^{*}\\ & +\left(0.05295+0.0007041T^{*}\right)\ln{\dot{\varepsilon }}^{*}] \end{array}$$

**Figure 11:**
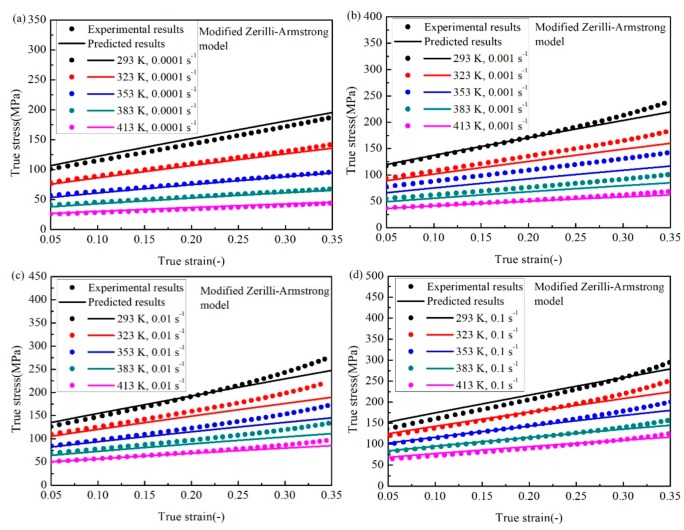
Comparison between the experimental data and predicted values by the modified Z–A model at the strain rate of (**a**) 0.0001 s^−1^, (**b**) 0.001 s^−1^, (**c**) 0.01 s^−1^, and (**d**) 0.1 s^−1^.

Similarly, Equation (14) can be used to predict the flow stress of the SnSbCu alloy. The comparison between the predicted data and experimental results is shown in Figure 11. As shown in Figure 11a–d, the accuracy of the modified Z–A model is better than the J–C model, especially at the low strain. While at high strains, the deviations between the predicted results and the experiments are still large.

### 3.4. Arrhenius-Type Model

The A-type constitutive model is another common model to predict the flow behavior of the metal materials under a wide range of temperatures and strain rates. The model consists of two equations including Equation (15), which means the Zener–Hollomon parameter representing the effects of the strain rate and temperature on deformation behaviors [49], and Equation (16) (i.e., Arrhenius equation [50]) describing the relation among the strain rate, stress, and temperature.

(15) $$Z=\dot{\varepsilon }\exp\left(\frac{Q}{RT}\right)$$

(16) $$\dot{\varepsilon }=AF\left(\sigma \right)\exp\left(-\frac{Q}{RT}\right)$$

where $\dot{\varepsilon }$ is the strain rate, *Q* is the activation energy of deformation, *R* is the universal gas constant (*R* = 8.31 J·mol^−1^·K^−1^), *T* is the experimental temperature, and *A* is the material constant. Furthermore, *σ* is the flow stress and *F*(*σ*) is a function about *σ*, as shown in Equation (17).

(17) $$F\left(\sigma \right)=\left\{ \begin{array}{ll} \sigma^{n_{1}}, & \alpha \sigma <0.8\\ \exp\left(\beta \sigma \right),\; & \alpha \sigma >1.2\\ \sinh{\left(\alpha \sigma \right)}^{n}, & \mathrm{for}\;\mathrm{all}\;\sigma \end{array}\right.$$

In this case, *n*_1_, *α*, and *β* are also material constants.

Under a certain temperature, substituting the expressions of *F*(*σ*) at low stress (*ασ* < 0.8) and high stress (*ασ* > 1.2) into Equation (16), the following equations could be derived.

(18) $$\dot{\varepsilon }=B\sigma^{n_{1}}$$

(19) $$\dot{\varepsilon }=C\exp\left(\beta \sigma \right)$$

where *B* and *C* are material constants. Taking the natural logarithm of the two sides of Equation (18) and Equation (19), two equations can be adopted.

(20) $$\ln\sigma =\frac{1}{n_{1}}\ln\dot{\varepsilon }-\frac{1}{n_{1}}\ln B$$

(21) $$\sigma =\frac{1}{\beta }\ln\dot{\varepsilon }-\frac{1}{\beta }\ln C$$

In this study, 15 different strains (0.05, 0.075, 0.1, 0.125, 0.15, 0.175, 0.2, 0.225, 0.25, 0.275, 0.3, 0.325, 0.35, 0.375, and 0.4) were selected. The figures about the strain of 0.3 are taken as examples to reveal the process of solving the material constants. According to Equations (20) and (21), it is clear that the values of *n*_1_ and *β* can be obtained from the curve of lnσ vs. $\ln\dot{\varepsilon }$ and the curve of σ vs. $\ln\dot{\varepsilon }$, respectively.

**Figure 12:**
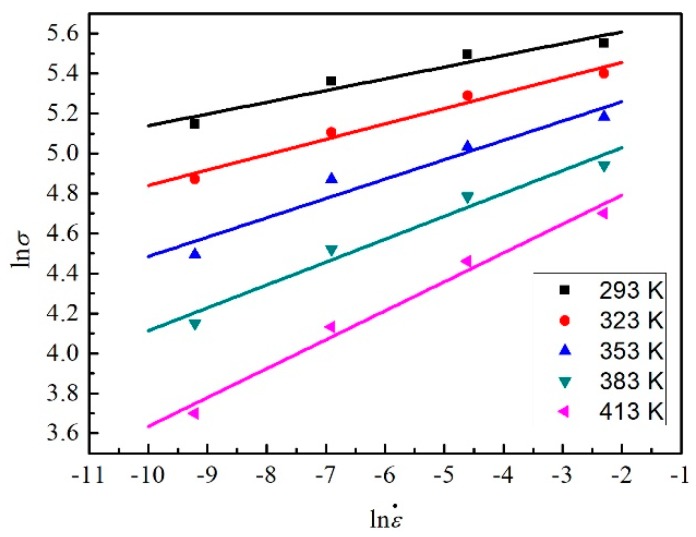
The relation between ln*σ* and $\ln\dot{\varepsilon }$ for five temperatures at the strain of 0.3.

As shown in Figure 12, the slopes of the lines under a different temperature can be obtained by the liner fit method. Due to the similar slopes, the mean value is taken as the value of 1/*n*_1_.

**Figure 13:**
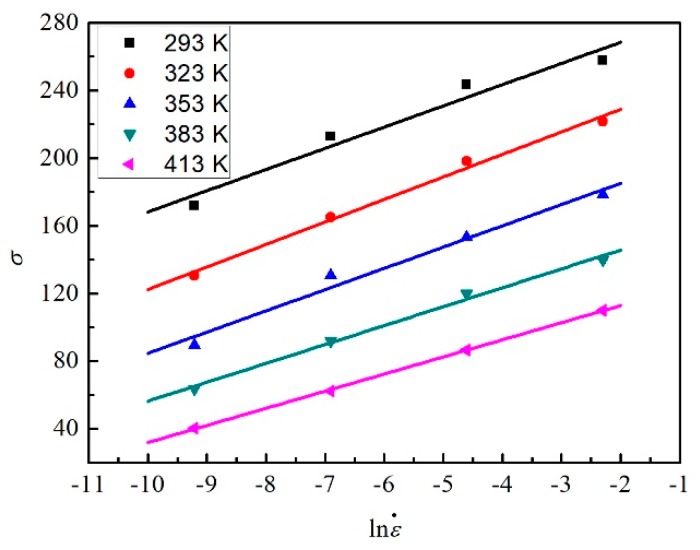
The relation between *σ* and $\ln\dot{\varepsilon }$ for five temperatures at the strain of 0.3.

Similarly, the value of 1/*β* can be calculated from Figure 13, which shows the relation between *σ* and $\ln\dot{\varepsilon }$. Therefore, the values of 1/*n*_1_ and 1/*β* are 0.09835 and 11.9474 separately. Furthermore, $\alpha =\beta /n_{1}=0.008232$.

Then, the expression of *F*(*σ*) for all *σ* (Equation (17)) can be substituted into Equation (16), which gives the equation below.

(22) $$\dot{\varepsilon }=A{\left[\sinh\left(\alpha \sigma \right)\right]}^{n}\exp\left(-\frac{Q}{RT}\right)$$

Taking the natural logarithm of both sides of Equation (22).

(23) $$\ln\left[\sinh\left(\alpha \sigma \right)\right]=\frac{\ln\dot{\varepsilon }}{n}+\frac{Q}{nRT}-\frac{\ln A}{n}$$

**Figure 14:**
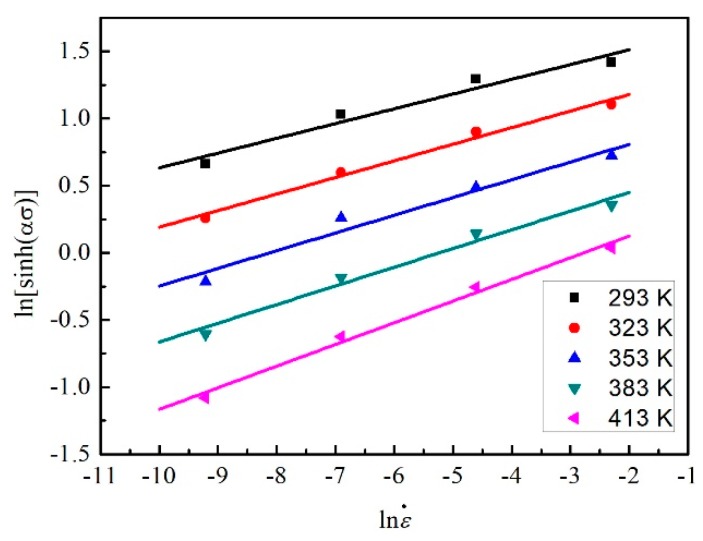
The relation between ln[sinh(*ασ*)] and $\ln\dot{\varepsilon }$ at the strain of 0.3.

**Figure 15:**
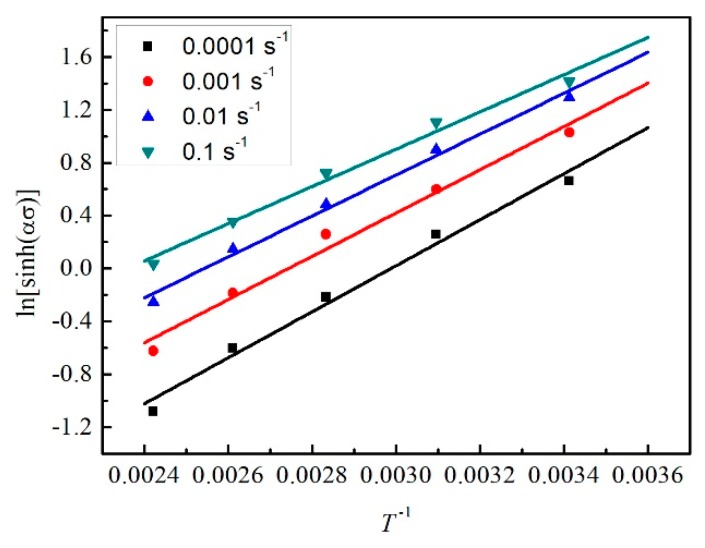
The relation between ln[sinh(*ασ*)] and 1/*T* at the strain of 0.3.

In addition, 1/*n* is the slope of the curve of ln[sinh(*ασ*)] vs. $\ln\dot{\varepsilon }$, and *Q*/*nR* is the slope of the curve of ln[sinh(*ασ*)] vs. 1/*T*. As shown in Figure 14 and Figure 15, the stress under five different temperatures (293 K, 323 K, 353 K, 383 K, and 413 K) and four strain rates (0.0001 s^−1^, 0.001 s^−1^, 0.01 s^−1^, and 0.1 s^−1^) were used to fit lines at the strain of 0.3. Figure 14 takes $\ln\dot{\varepsilon }$ as the abscissa and five lines at different temperatures are fitted. Differently, Figure 15 takes 1/*T* as the abscissa and four lines at different strain rates are fitted. As a result, the value of *n* can be obtained first and then *Q* can be calculated. The intercepts of fitting lines in Figure 14 are the values of *Q*/(*nRT*) − ln*A*/*n* at different temperatures. Substituting *Q* and *n* into the expression and averaging, ln*A* can be calculated. At the strain of 0.3, *n* = 7.5078, *Q* = 98.742 kJ/mol, and ln*A* = 26.0415.

Like the calculation process of *σ*, *n*, *Q,* and ln*A* at the strain of 0.3, the other 14 sets of material constants can be acquired at different strains. However, the values of each constant vary greatly with the raise of the strain. Four 5th order polynomials, as shown in Equation (24), were used to describe the effect of true strain on the material constants.

(24) $$\begin{array}{l} \alpha =\alpha_{0}+\alpha_{1}\varepsilon +\alpha_{2}\varepsilon^{2}+\alpha_{3}\varepsilon^{3}+\alpha_{4}\varepsilon^{4}+\alpha_{5}\varepsilon^{5}\\ n=n_{0}+n_{1}\varepsilon +n_{2}\varepsilon^{2}+n_{3}\varepsilon^{3}+n_{4}\varepsilon^{4}+n_{5}\varepsilon^{5}\\ Q=Q_{0}+Q_{1}\varepsilon +Q_{2}\varepsilon^{2}+Q_{3}\varepsilon^{3}+Q_{4}\varepsilon^{4}+Q_{5}\varepsilon^{5}\\ \ln A=A_{0}+A_{1}\varepsilon +A_{2}\varepsilon^{2}+A_{3}\varepsilon^{3}+A_{4}\varepsilon^{4}+A_{5}\varepsilon^{5} \end{array}$$

**Figure 16:**
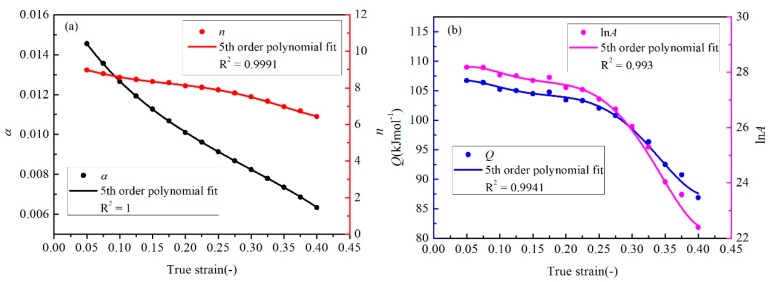
The variation of (**a**) *α* and *n*, (**b**) *Q* and ln*A* at 15 true strains fit with the fifth order polynomial.

As shown in Figure 16, four fitting lines about the material constants exhibit good correlation and adaptability. The pending coefficients of each curve are written in Figure 16, and all values of *R*^2^ are bigger than 0.99. Furthermore, the coefficients of Equation (24) are calculated in Table 2.

Lastly, the determinate material constants (i.e., *σ*, *n*, *Q*, and ln*A*) were used to predict the flow behavior of the SnSbCu alloy. According to a hyperbolic sine function, the constitutive model about the influence of the Zener–Hollomon parameter and coefficient on the flow behavior can be written as the equation below [51].

(25) $$\sigma =\frac{1}{\alpha }\ln\left\{{\left(\frac{Z}{A}\right)}^{1/n}+{\left[{\left(\frac{Z}{A}\right)}^{2/n}+1\right]}^{1/2}\right\}$$

**Figure 17:**
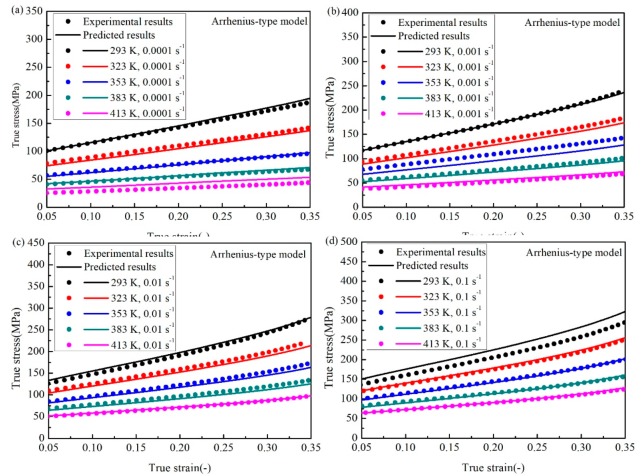
Comparison between the experimental data and predicted values by the A-type model at the strain rate of (**a**) 0.0001 s^−1^, (**b**) 0.001 s^−1^, (**c**) 0.01 s^−1^, and (**d**) 0.1 s^−1^.

Using Equation (25), the comparisons between the experimental and predicted results at different temperatures and strain rates are shown in Figure 17. It is observed that the predicted results are in good agreement with the experimental results from the low strain to high strain under all focused temperatures and strain rates.

### 3.5. Accuracy Analysis

Based on the results above, the predictive ability of the three models is generally understood, but it needs to be carefully analyzed to determine which model is suitable for the SnSbCu alloy. Therefore, the accuracy of the constitutive model should be concretely displayed. The correlation coefficient (*R*) and average absolute relative error (*AARE*) are chosen to compare the experimental results and predicted results from models. *R* means the strength of linear relation between the two sets of data, while *AARE* is an unbiased statistical parameter to determine the predictability of models through a term by term. The expressions of *R* and *AARE* are as follows.

(26) $$R=\frac{\sum_{i=1}^{i=N}\left(\sigma_{e}^{i}-{\bar{\sigma }}_{e}\right)\left(\sigma_{p}^{i}-{\bar{\sigma }}_{p}\right)}{\sqrt{\sum_{i=1}^{i=N}{\left(\sigma_{e}^{i}-{\bar{\sigma }}_{e}\right)}^{2}\sum_{i=1}^{i=N}{\left(\sigma_{p}^{i}-{\bar{\sigma }}_{p}\right)}^{2}}}$$

(27) $$AARE\left(\%\right)=\frac{1}{N}\sum_{i=1}^{i=N}\left|\frac{\sigma_{e}^{i}-\sigma_{p}^{i}}{\sigma_{e}^{i}}\right|\times 100$$

where $\sigma_{e}^{i}$ and $\sigma_{p}^{i}$ are the experimental plastic flow stress data and predicted results from the models, respectively, at the same strains for different strain rates and temperatures. ${\bar{\sigma }}_{e}$ represents the average of $\sigma_{e}^{i}$ and ${\bar{\sigma }}_{p}$, which is that of $\sigma_{p}^{i}$. *N* is the number of data points selected at the plastic process. In the study, 742 data points from experiments were used for data analysis.

**Figure 18:**
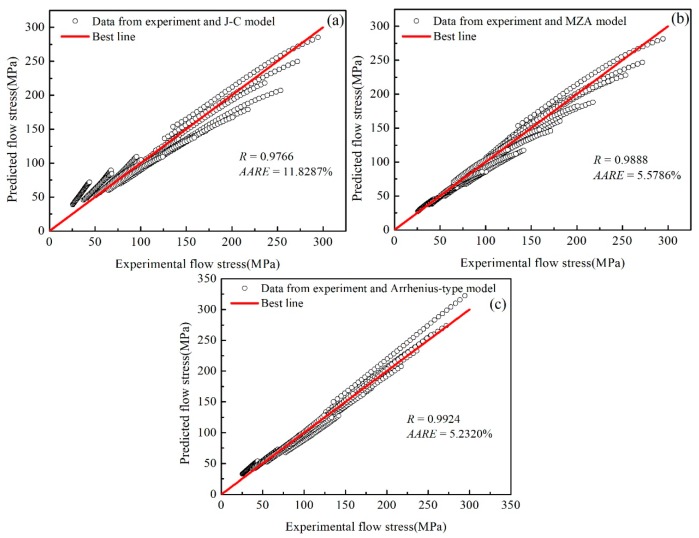
Comparison between experimental results and predicted results from (**a**) the J–C model, (**b**) the modified Z–A model, and (**c**) the A-type model for different strain rates and temperatures.

As shown in Figure 18a–c, it is observed that most data points are close to the best line among these models. The value of *R* of the A-type model is the biggest at 0.9924, which is followed by the modified Z–A model (0.9888). The correlation coefficient of the J–C model is the smallest, at 0.9766 (>0.95). Regarding the *AARE*, the value of the J–C model (11.8287%) is more than twice as big as the other two models. The *AARE* percentage of the modified Z–A model (5.5786%) is close to that of the A-type model (5.2114%).

From Figure 18, data points are composed of 20 sets of data for different strain rates and temperatures. In Figure 18a,b, each set of data points is presented as a nonlinear line. At low stress and strain, the data points are above the best line. With the increase of stress and strain, the data points gradually move below the best line. Compared with the trends of the J–C model and the modified Z–A model, the slope of the line constituted by each set of data points from the A-type model is similar with that of the best line. That means the A-type model can track the flow behavior of the SnSbCu alloy and predict the stress at wide ranges of the strain, strain rate, and temperature. Additionally, with the variety of strain, the error from the other two prediction models would become bigger with the increase of strain. Furthermore, the influence of the strain, strain rate, and temperature on the flow stress is highly nonlinear. The three factors would affect each other, which decreases the accuracy of a traditional prediction model and the prediction range of the strain, strain rate, and the temperature become limited.

**Figure 19:**
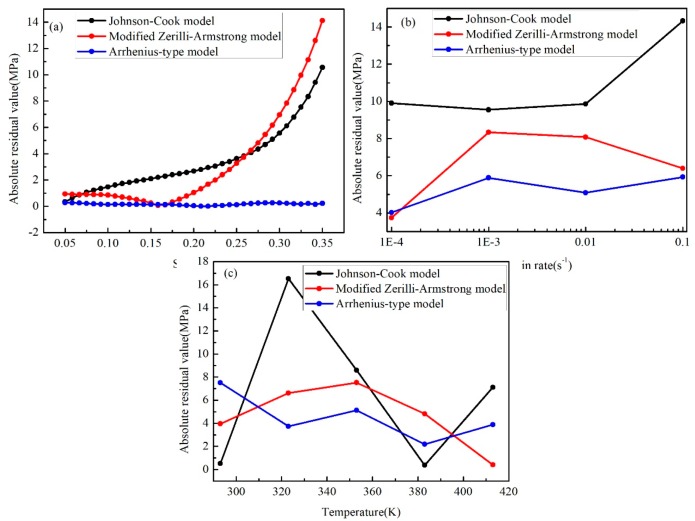
Absolute residual values from three models of (**a**) strain, (**b**) strain rate, and (**c**) temperature.

To explain the nonlinear degree of effect of the three factors (strain, strain rate, and temperature) for the flow stress on the fitting results in different models, the absolute residual values for the factors are drawn in Figure 19. As shown in Figure 19a, it is clear that the residual values increase with the raise of strain for the J–C model and the modified Z–A model, while the value for the A-type model fluctuates at about zero. Regarding the strain rate (Figure 19b) similarly, the absolute residual value for the A-type model is the smallest and the values for the other two models vary more than the A-type model at the strain rate from 0.0001 s^−1^ to 0.1 s^−1^. According to Figure 19c, the value obtained from the J–C model changes largely from 293 K to 413 K. The trends of the other two model are similar. However, the value for the A-type model is smaller than the one from the modified Z–A model.

Above all, it has been found in this study that the J–C model can predict the flow behavior of the SnSbCu alloy at the condition close to the reference strain rate and temperature. However, it is disabled to predict the flow behavior of the SnSbCu alloy over the wide range of strain rates and temperatures. This is because the J–C model lacks the interaction among the strain, strain rate, and temperature. Compared with the J–C model, the modified Z–A model has better accuracy at low strains, while it could not track the flow behavior very well, which means that the accuracy at the high strain is not as good as that at the low strain (shown in Figure 11). The A-type model can treat the nonlinear relation among the factors well over the whole range of strains, which seems to be the most suitable prediction model for the SnSbCu alloy (shown in Figure 18).

In addition, it should be noted that there are five material constants in the J–C model and seven in the modified Z–A model. The number of material constants in the A-type constitutive model is 24, which is several times that of the other two models. This is a reason why the A-type model has higher accuracy. Figuring out all material constants of the A-type model costs several times longer than that for two other models. Thus, for the engineering practice, a balance between the efficiency and accuracy should be considered. For some numerical analysis, which focus on the accuracy, although the A-type model need more experimental data and time to calculate material constants, the accuracy of this model makes up its defects.

## 4. Conclusions

Obtaining the accurate constitutive model of the materials would be helpful to develop more efficient and precise numerical models for analyzing the tribological behavior further in the field focused by the authors. Therefore, in this study, the flow behavior of the SnSbCu alloy was studied through compression tests in temperatures ranging from 293 to 413 K and a strain rate of 0.0001–0.1 s^−1^. Three current constitutive models, the J–C, the modified Z–A, and the A-type models, were used to describe its flow behavior, and the predicted results given by these three models were compared with the experiments. Several conclusions were drawn, which are shown below.

1. The strain rate hardening effect and the temperature softening effect were notable for the flow behavior of the SnSbCu alloy.
2. The J–C model could describe the flow behavior in the reference strain rate and temperature case, while, for other cases, the description was not effective since this model lacked the interaction of the strain rate hardening effect and the temperature softening effect.
3. The prediction of the modified Z–A model could match the experimental results effectively at a low strain. However, the errors between experimental and predicted results enlarged with the increase of the strain.
4. The A-type constitutive model can predict the flow behavior of the material under the whole focused range of temperatures and strain rates with the smallest errors and largest correlation coefficient among the three models, since the Zener–Hollomon parameter was employed to describe the interaction of the effect of the strain rate and the deformation.

## Figures and Tables

**Table 1:** The value of *S*_1_ and *I*_1_ for 15 strains.

Strain (–)	*I*_1_	*S*_1_
0.050	4.6752	−0.01133
0.075	4.7444	−0.01149
0.100	4.8044	−0.01159
0.125	4.8626	−0.01167
0.150	4.9166	−0.01172
0.175	4.9691	−0.01179
0.200	5.0184	−0.01184
0.225	5.0653	−0.01189
0.250	5.1106	−0.01194
0.275	5.1535	−0.01200
0.300	5.1951	−0.01205
0.325	5.2365	−0.01209
0.350	5.2785	−0.01212
0.375	5.3243	−0.01212
0.400	5.3738	−0.01206

**Table 2:** The value of each coefficient in the expression of material constants.

*α*	*n*	*Q*(kJ/mol)	ln*A*
*α*_0_ = −0.9000	*n*_0_ = +1433	*Q*_0_ = +4.318 × 10^4^	*A*_0_ = +1.332 × 10^4^
*α*_1_ = +1.1240	*n*_1_ = −1240	*Q*_1_ = −4.375 × 10^4^	*A*_1_ = −1.364 × 10^4^
*α*_2_ = −0.6547	*n*_2_ = +274.9	*Q*_2_ = +1.537 × 10^4^	*A*_2_ = +4880
*α*_3_ = +0.2269	*n*_3_ = +6.240	*Q*_3_ = −2.353 × 10^3^	*A*_3_ = −776.4
*α*_4_ = −0.0619	*n*_4_ = −11.46	*Q*_4_ = +1.351 × 10^2^	*A*_4_ = +50.14
*α*_5_ = +0.0172	*n*_5_ = +9.510	*Q*_5_ = +1.042 × 10^2^	*A*_2_ = +27.09
